# Effects of beta-conglycinin intake on circulating FGF21 levels and brown adipose tissue activity in Japanese young men: a single intake study and a randomized controlled trial

**DOI:** 10.1186/s40101-020-00226-w

**Published:** 2020-07-22

**Authors:** Hirokazu Taniguchi, Keigo Shimizu, Sayori Wada, Shinsuke Nirengi, Haruki Kataoka, Akane Higashi

**Affiliations:** 1 Division of Applied Life Sciences, Graduate School of Life and Environmental Sciences, Kyoto Prefectural University, 1-5 Hangi-cho, Shimogamo, Sakyo-ku, Kyoto, 606-8522 Japan; 2 Faculty of Agriculture, Ryukoku University, Otsu, Shiga 520-2194 Japan; 3 Clinical Research Institute, Division of Preventive Medicine, National Hospital Organization Kyoto Medical Center, Kyoto, 612-8555 Japan

**Keywords:** Fibroblast growth factor 21, Thermogenesis, Soy protein, Thermography, RCT

## Abstract

**Background:**

Human brown adipose tissue (BAT) activity has beneficial effects on body composition and glucose metabolism. A previous study reported that beta-conglycinin intake induced postprandial fibroblast growth factor 21 (FGF21) secretion, thereby promoting adipose tissue thermogenesis in mice. Since it has not been evaluated whether beta-conglycinin intake is associated with induced FGF21 secretion and BAT thermogenesis in humans, the current study examined the effects of beta-conglycinin intake on circulating FGF21 level and BAT activity.

**Methods:**

Twenty-two healthy young male subjects participated. This study consisted of 2 interventional studies. In one of them, the effects of single beta-conglycinin intake at thermoneutral temperature on circulating FGF21 levels were examined (*n* = 7). The other study was a single-blinded randomized crossover trial of 2 weeks (*n* = 14). The subjects were exposed to mild cold conditions using a climatic chamber, and BAT activity was analyzed using thermography. Serum FGF21 level was determined by ELISA in these studies.

**Results:**

In the single intake study, serum FGF21 level was the highest before beta-conglycinin intake and gradually and significantly decreased throughout the 2-h experimental period (*P* < 0.05). The randomized crossover trial showed that 2-week beta-conglycinin intake did not affect serum FGF21 level and BAT activity, whereas changes (Δ) in baseline levels of serum FGF21 were positively correlated with Δ BAT activity (*P* < 0.05). In addition, analysis of each group revealed that there was significant correlation between the Δ serum FGF21 level and Δ BAT activity in the beta-conglycinin group (*P* < 0.05), but not in the placebo group.

**Conclusions:**

This study reveals that although serum FGF21 levels are not increased by a single or short-term intake of beta-conglycinin, the Δ basal FGF21 level is associated with Δ BAT activity. These results suggest that human FGF21 responsiveness is different from that of rodents and support the importance of FGF21 in human BAT thermogenesis.

**Trial registration:**

This study is registered with University Hospital Medical Information Network in Japan (number 000038723, https://upload.umin.ac.jp/cgi-open-bin/ctr_e/ctr_view.cgi?recptno=R000043942).

## Background

Brown adipose tissue (BAT) is characterized by a high expression of the mitochondrial uncoupling protein 1, which enhances thermogenic energy expenditure [1, 2]. It has been demonstrated that human BAT has beneficial effects on body composition and glucose metabolism, and the same is true for animals [1–5]. Previous studies have undertaken the strategy of increasing BAT activity through dietary intake activating the sympathetic nervous system [6, 7]. It is therefore necessary to further elucidate the regulatory factors of human BAT activity.

Fibroblast growth factor 21 (FGF21) is a hormonal factor that is primarily secreted by the liver and improves whole-body metabolism [8–11]. Previous animal studies have shown that FGF21 is related to an increase in browning of the white adipose tissue [12, 13], and, according to some studies, activating of the BAT [12, 14]. Human studies have also reported beneficial effects of FGF21. For instance, ingestion of FGF21 or an analog has been shown to improve glycolipid metabolism [15–17]. Moreover, circulating FGF21 levels correlated with the BAT activity during cold exposure in male subjects [18, 19]. It is considered that the increase in circulating FGF21 stimulates BAT thermogenesis. In contrast, in obese rodents and humans, circulating FGF21 is chronically elevated [20, 21], which has been characterized as a FGF21-resistant state [21]. Although these results suggest that basal FGF21 concentration may relate to the thermogenic impairment of the adipose tissue, the association between baseline changes in circulating FGF21 levels and BAT activity has not been examined.

In a previous study, single ingestion of beta-conglycinin, a protein isolated from soy beans, induced postprandial FGF21 secretion in mice [22]. Furthermore, long-term beta-conglycinin intake suppressed high resting level of circulating FGF21, induced adipose tissue thermogenesis, and prevented body fat accumulation in mice fed high fat diets [22, 23]. In addition, interventional human studies have demonstrated that beta-conglycinin intake improves metabolic disorders including dyslipidemia [24] and visceral fat obesity [25]. It is therefore likely that the favorable mechanism of beta-conglycinin intake may be related to BAT activation by FGF21. However, it has not been evaluated whether beta-conglycinin intake is associated with induced FGF21 secretion and BAT thermogenesis in humans.

The purpose of this study was to identify the effects of beta-conglycinin intake on circulating FGF21 levels and BAT activity. This study initially determined whether a single intake of beta-conglycinin affects circulating FGF21 levels in healthy young men. Thereafter, we performed a randomized controlled trial (RCT) to examine the effects of 2-week beta-conglycinin intake on circulating FGF21 concentration and BAT activity.

## Methods

### Subjects

Twenty-two healthy young male subjects (age 19–22 years) participated in this study. The subjects were recruited personally and through a volunteer club in the Kyoto Prefectural University. The inclusion criteria were being male and having an age of 18–22 years. The exclusion criteria included a history of chronic disease and intake of medication. All participants provided written informed consent before enrolment in the study, which was approved by the ethical committee of the Kyoto Prefectural University (Approved number 175). This study is registered with UMIN (University Hospital Medical Information Network in Japan), number 000038723.

### Study design

**Fig 1.**
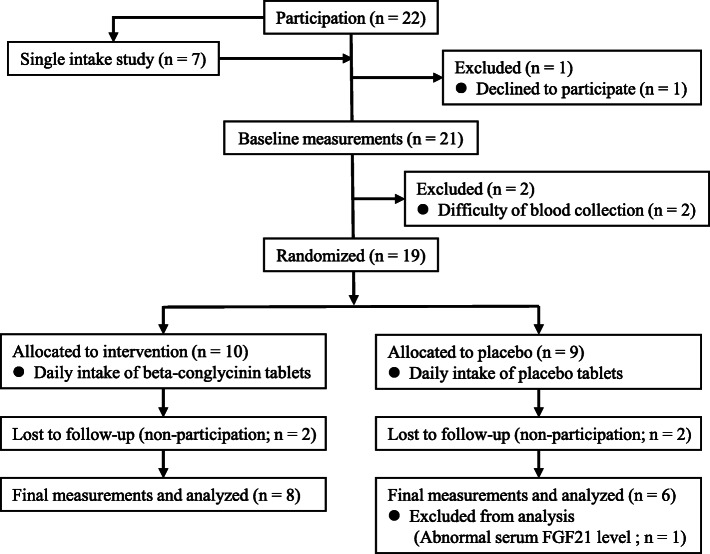
Flow diagram of the participants in the study

As shown in Fig. 1, this study consisted of 2 interventional studies performed in the winter of 2019.

The first study examined the effects of single beta-conglycinin intake on circulating FGF21 levels without cold exposure. Seven subjects were selected. The single intake study started at 9:00 AM after an overnight fast, and the subjects were seated at rest during the experiment. After resting for 30 min, baseline blood samples were collected, and then subjects took beta-conglycinin tablets containing 10.6 g protein including 9.2 g beta-conglycinin (20 tables of WELLBEST beta-conglycinin; Fuji Industry, Tokyo, Japan). The amount of beta-conglycinin was more than that in previous interventional studies, which examined the effects of beta-conglycinin intake on acute appetite sensation [26], lipid metabolism [24], and visceral obesity [25]. After ingestion, blood samples were collected at 1 h and 2 h after the beta-conglycinin intake. The room temperature was maintained at 24 °C ± 2 °C throughout this study.

The other study was a single blind RCT. One subject did not participate due to lack of time before allocation, and thus 21 subjects were allocated into the beta-conglycinin group (*n* = 10) or the placebo group (*n* = 11). Baseline measurements were performed to determine anthropometric characteristics, blood biochemical parameters, daily food intake, and BAT activity. After baseline measurements, 2 subjects from the placebo group were excluded because of difficulties in collecting blood samples due to vasovagal reaction or rough skin surface. The nutritional composition of beta-conglycinin and placebo tablets has been shown in Table 1. Subjects were instructed to take either 20 tablets of WELLBEST beta-conglycinin (Fuji Industry, Tokyo, Japan) in the beta-conglycinin group or 20 tablets of placeplus (Placebo pharmaceutical company, Otsu, Japan) in the placebo group daily. Both groups were instructed to take 10 tablets in the morning and 10 tablets in the evening. All subjects were free-living and were instructed to consume their habitual diet throughout the study, and final measurements were performed after the 2-week interventional period.

**Table 1:** Nutritional composition of beta-conglycinin and placebo tablets

	Beta-conglycinin	Placebo
Energy (kcal)	64.0	16.0
Protein (g)	10.6	0.0
Beta-conglycinin (g)	9.2	0.0
Fat (g)	0.2	0.1
Carbohydrate (g)	7.8	3.8

Twenty tablets composition was shown that it is used in single intake study and as amounts of daily intake in randomized controlled trials.

### Baseline and final measurements in the randomized crossover trial

Mild-cold exposure was performed to evaluate BAT activity and serum FGF21 level in the randomized crossover trial. Subjects entered the climatic chamber (TBRR-9A4GX; ESPEC, Osaka, Japan) at 9:00 AM after an overnight fast, and the initial temperature of the climatic chamber was set at 27 °C. They were seated at rest for 30 min, and then baseline body surface temperature was measured as described below. After blood samples were collected, the temperature of the climatic chamber was reduced to 19 °C. The changes in chamber temperature were done within 5 min, and the cold exposure started at 19 °C in the chamber [27, 28]. Thereafter, both body surface temperature and blood samples were collected at 1 h and 2 h of mild-cold exposure. Before and during cold exposure, subjects were asked to rate shivering, cold sensation, and discomfort using a visual analog scale [27].

### Measurement of BAT activity

BAT activity was determined as per a previously described method [27]. In brief, body surface temperature was measured using a thermal imaging camera (DE-TC1000T; D-eyes, Osaka, Japan). The supraclavicular temperature adjacent to BAT location on both the right and left sides was measured from each image. Chest temperature was simultaneously measured as a control for underlying BAT depots. The images of body surface temperature were analyzed using a modified (D-eyes) version of the Thermal-Cam v.1.1.0.9 software (Laon People, Seoul, Korea), and the average of supraclavicular temperature minus chest temperature was estimated as BAT activity. In the previous study, the thermography index was correlated (*r* = 0.74, *P* < 0.05) using ^18^F fluorodeoxyglucose-positron emission tomography/computed tomography, a standardized method for measuring BAT activity in humans [27].

### Blood analysis

Blood samples were centrifuged at 3000*g* for 5 min. Serum was stored at – 80 °C until the time of analysis. The concentrations of serum glucose and triglycerides and the serum enzymatic activities of aspartate aminotransferase (AST) and alanine aminotransferase (ALT) were measured using a Fuji Dry Chem 4000 analyzer (Fuji film, Tokyo, Japan). The serum FGF21 concentration was determined using a commercially available enzyme-linked immunosorbent assay (ELISA) kit (DF2100; R&D Systems, Minneapolis, MN, USA) according to the manufacturer’s instructions. The intra- and inter-assay coefficients of variation reported by the manufacturer were 2.9–3.9% and 5.2–10.9%, respectively.

### Anthropometric characteristics and dietary food intake

Body weight and body fat percentage were measured using an electronic scale (V-body HBF-359; Omron, Kyoto, Japan), and body mass index (BMI) was calculated from measurements of height and body weight. Energy, protein, carbohydrate, and fat intake were also assessed using a Food Frequency Questionnaire based on the food groups (FFQg Ver 5.0; Kenpakusha, Tokyo, Japan), which estimates habitual intake by portion size and food frequency [29]. Supplemental nutrients from beta-conglycinin and placebo tablets were added to nutritional intake in the final measurements of the RCT.

### Statistical analysis

All statistical analyses were performed using SPSS, version 24.0 (SPSS, Chicago, Illinois, USA). The Kolmogorov-Smirnov test was performed to assess the normality of data distribution. The one-way ANOVA and the post hoc Tukey’s HSD test were used to evaluate the difference in serum FGF21 levels between before, 1 h after, and 2 h after beta-conglycinin intake in the single intake study. Baseline characteristics and changes in variables during the randomized crossover trial were compared using the Student’s *t* test. Two-way ANOVA analysis (time × group) was used to determine absolute value difference between the groups. Relationships between changes in all variables were determined by the Pearson’s correlation coefficients. All measurements and calculated values have been presented as the mean ± SD, and the level of statistical significance was set at *P* < 0.05.

## Results

**Fig 2.**
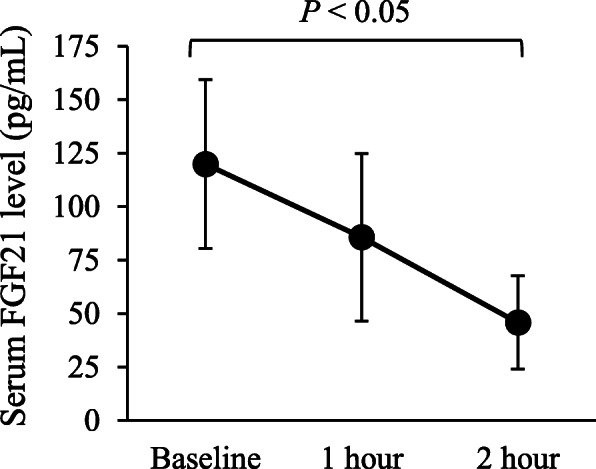
Effects of a single intake of beta-conglycinin on serum FGF21 levels in young male subjects. FGF21, fibroblast growth factor 21

In the single intake study, the participating male subjects were young (21.1 years ± 0.7 years) and had a normal BMI (20.1 kg/m^2^ ± 1.4 kg/m^2^). The ELISA assay revealed that serum FGF21 level was the highest before beta-conglycinin intake and gradually decreased after single ingestion in the thermoneutral room (Fig. 2). One-way ANOVA revealed that the serum FGF21 levels were significantly lower 2 h after beta-conglycinin intake when compared to the baseline measurement (*P* < 0.05).

Table 2 summarizes the baseline characteristics of the subjects and the changes in variables during the 2-week randomized controlled trial. The 14 young male subjects had normal BMI, serum metabolic parameters, and nutrient intake. Four subjects did not participate in the final measurements, and 1 subject was excluded because of an abnormal increase in serum FGF21 concentration (+ 509.7 pg/mL during the interventional period) that was more than 5 SD above the mean of the changes in the placebo group. Therefore, the results of 14 subjects were analyzed in total. The final number of subjects was 8 in the beta-conglycinin group and 6 in the placebo group. At baseline measurement, the height was significantly higher in the placebo group compared to the beta-conglycinin group (*P* < 0.05), whereas there was no statistical significance in other variables between the groups. The 2-week interventional study did not show a statistical difference in any variable including anthropometric characteristics, dietary food intake, and serum biochemical variables between the beta-conglycinin and placebo groups (Table 2).

**Table 2:** Changes in the variables during 2-week beta-congllycinin and placebo intakes

	Beta-conglycinin group (*n* = 8)									Placebo (*n* = 6)									*P*
	Pre			Post			⊿			Pre			Post			⊿			
Age (year)	20.9	±	1.2							21.3	±	0.7							
Height (cm)	168.9	±	4.7							176.3	±	3.7							
Weight (kg)	59.8	±	7.0	59.9	±	6.8	0.2	±	1.0	62.0	±	6.3	62.7	±	6.3	0.8	±	1.1	0.337
BMI (kg/m²)	20.9	±	2.3	21.0	±	2.3	0.1	±	0.3	20.0	±	1.7	20.1	±	1.6	0.0	±	0.3	0.769
Body fat (%)	14.1	±	4.6	14.3	±	5.0	0.3	±	1.4	11.6	±	5.2	11.9	±	5.2	0.3	±	1.1	0.906
Serum metabolic parameters																			
Glucose (mg/dL)	88.9	±	2.3	90.5	±	4.8	1.6	±	4.2	93.8	±	5.8	89.5	±	8.4	-4.3	±	6.8	0.071
Triglyceride (mg/dL)	52.3	±	23.4	69.8	±	23.5	17.5	±	23.5	52.2	±	25.7	65.0	±	29.9	12.8	±	25.3	0.738
AST (U/I)	20.4	±	2.9	20.6	±	2.9	0.3	±	2.9	20.0	±	2.4	19.8	±	1.8	-0.2	±	1.3	0.762
ALT (U/I)	21.4	±	16.2	16.1	±	3.5	-5.3	±	13.9	17.3	±	6.7	17.7	±	5.1	0.3	±	2.3	0.383
FGF21 (pg/mL)	146.6	±	85.0	158.1	±	91.6	11.4	±	85.1	68.0	±	29.4	84.0	±	52.2	16.1	±	53.3	0.913
Energy and nutrients intake (/day)																			
Energy (kcal)	2144	±	599	2137	±	465	-7	±	285	1899	±	220	2303	±	409	404	±	671	0.147
Protein (g)	67.1	±	20.1	89.4	±	16.9	22.3	±	17.7	70.2	±	11.6	78.7	±	11.5	8.5	±	16.6	0.182
Fat (g)	74.2	±	21.5	66.9	±	17.7	-7.2	±	14.1	66.7	±	7.6	86.0	±	24.8	19.2	±	34.6	0.075
Carbohydrate (g)	279.5	±	86.4	280.7	±	63.2	1.2	±	45.2	243.6	±	33.4	285.3	±	44.2	41.8	±	78.6	0.253
BAT activity (supraclavicular - chest skin temperature; °C)																			
1-h after cold exposure	0.89	±	0.28	0.86	±	0.34	-0.03	±	0.24	0.90	±	0.34	0.84	±	0.35	-0.08	±	0.13	0.656
2-h after cold exposure	0.87	±	0.31	0.91	±	0.38	0.04	±	0.28	0.77	±	0.37	0.88	±	0.39	0.08	±	0.23	0.804

BMI, body mass index; AST, aspartate aminotransferase; ALT, alanine aminotransferase; FGF21, fibroblast growth factor 21; BAT, brown adipose tissue. Values are presented as mean ± SD. P values were obtained by unpaired Student's t test

**Fig 3.**
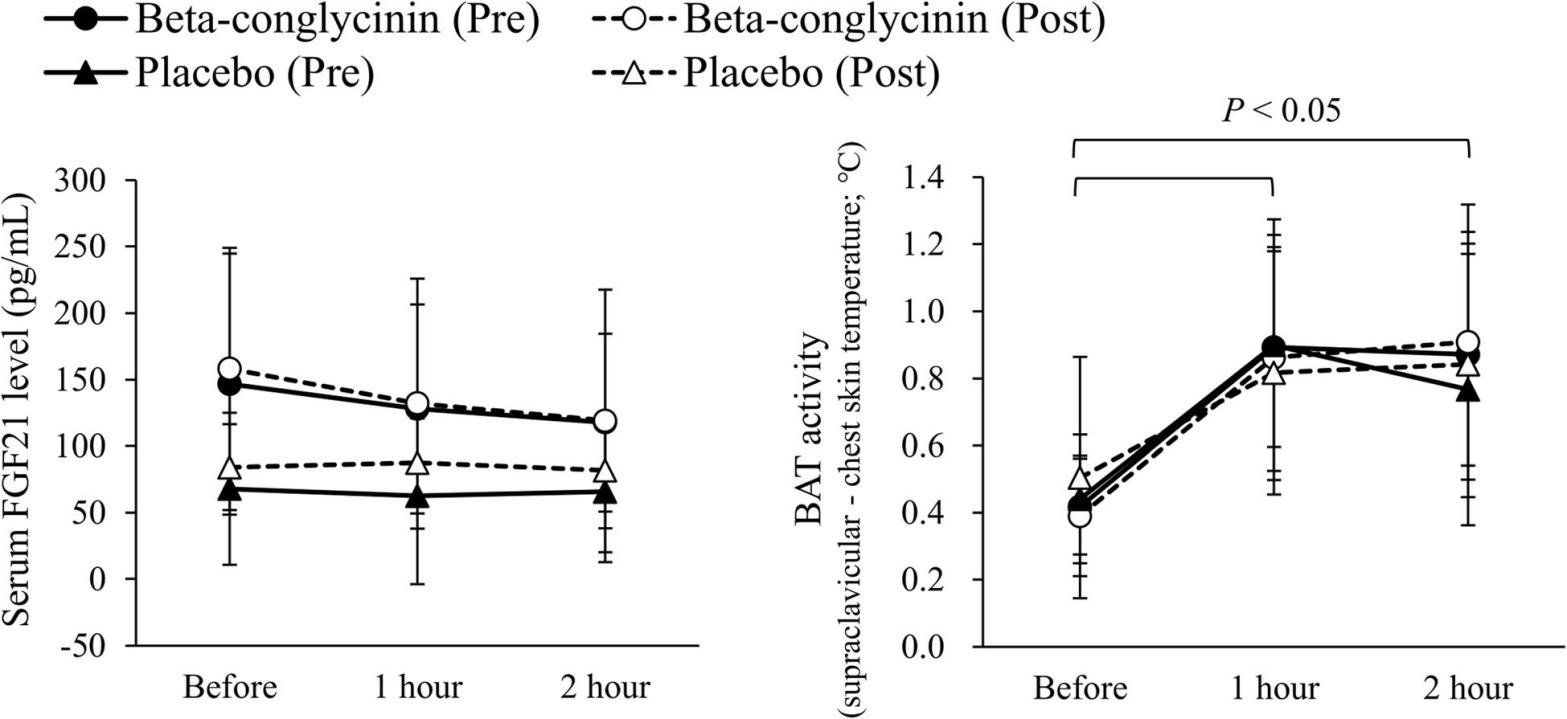
Serum FGF21 levels and BAT activity before and during cold exposure in the pre- and post-randomized controlled trials. FGF21, fibroblast growth factor-21; BAT, brown adipose tissue

As shown in Fig. 3, serum FGF21 concentration did not decrease during cold exposure, whereas BAT activity was significantly increased by cold exposure (*P* < 0.05). There was no significant difference in BAT activity between 1 h and 2 h after cold exposure, indicating that BAT activity had reached a plateau at 1 h cold exposure. The randomized controlled trial found that the absolute values of serum FGF21 concentration and BAT activity were not different between the two groups before and after intervention periods. Moreover, there were no significant changes in baseline serum FGF21 levels and BAT activity 1 h and 2 h after cold exposure between the beta-conglycinin and placebo groups (Table 2). Shivering response, cold sensation, and discomfort were not statistically different between the two groups in this study (data not shown).

**Fig 4.**
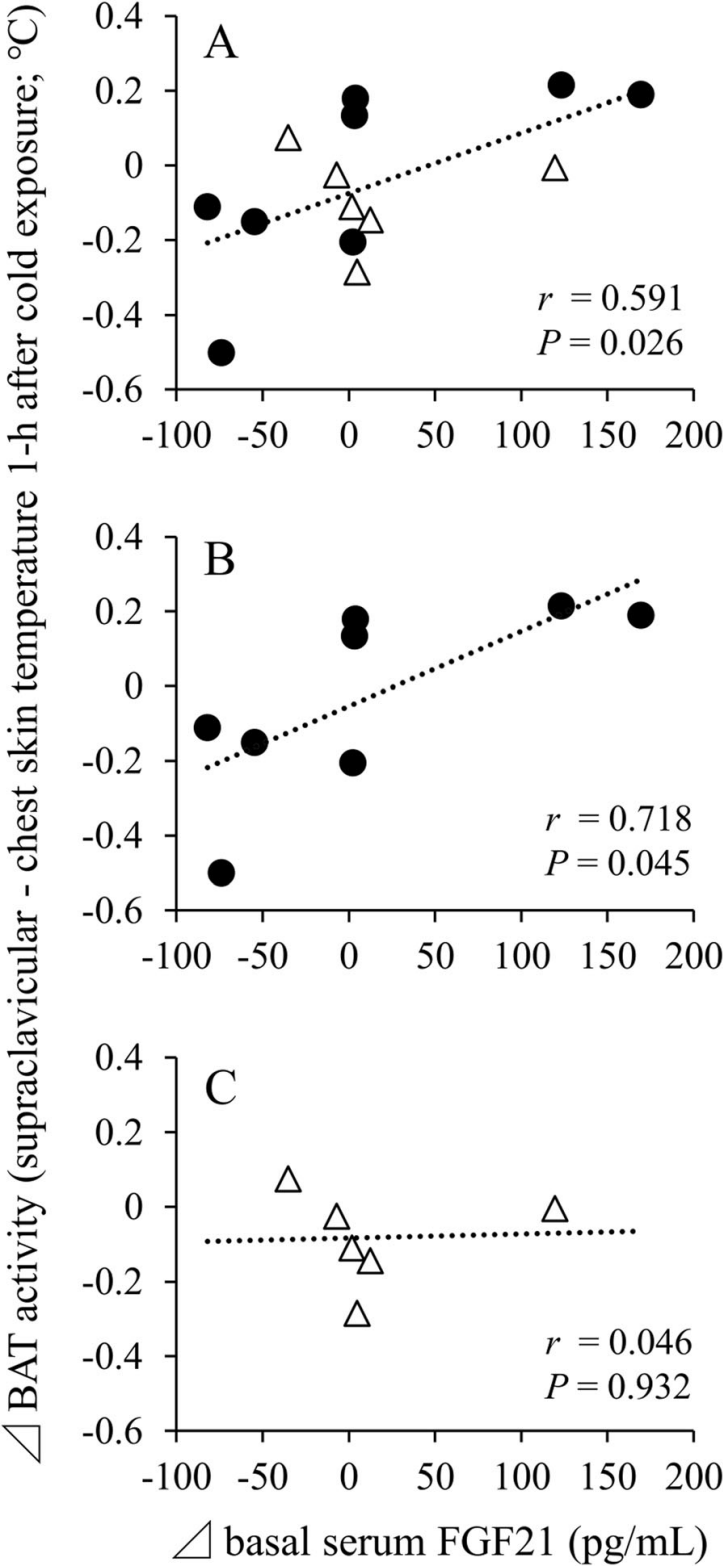
Correlations of the changes in the baseline serum FGF21 levels with BAT activity in young male subjects. Closed circle shows the beta-conglycinin group, and open triangle shows the placebo group. FGF21, fibroblast growth factor 21; BAT, brown adipose tissue

In turn, the determinants of BAT activity were examined using correlation analysis (Table 3). The length of cold exposure did not increase BAT activity, and serum FGF21 concentration remained steady during cold exposure in the interventional study. Therefore, the changes in BAT activity (Δ BAT activity) 1 h after cold exposure and the changes in baseline serum FGF21 level (Δ baseline FGF21 level) were used as variables. In all subjects, the Δ BAT activity was not associated with changes in anthropometric characteristics and serum biochemical variables, whereas Δ baseline FGF21 level was significantly and positively correlated with Δ BAT activity (Fig. 4a, *P* < 0.05). In addition, analysis of each group revealed that there was a significant correlation between the changes in Δ baseline FGF21 level and Δ BAT activity in the beta-conglycinin group (Fig. 4b, *P* < 0.05), but not in the placebo group (Fig. 4c). Although there was no significant correlation in all subjects, the changes in dietary fat amount (Δ fat intake) were significantly and negatively correlated with Δ BAT activity in the beta-conglycinin group (Table 3, *P* < 0.05).

**Table 3:** Correlations between BAT activity and other variables in young male subjects

	Dependent variable: Δ BAT activity (1-h after cold exposure)					
	Total (*n* =14)		Beta-conglycinin (*n* =8)		Placebo (*n* = 6)	
	*r*	*P*	*r*	*P*	*r*	*P*
Δ BMI (kg/m²)	0.356	0.211	0.335	0.417	0.430	0.394
Δ Body fat (%)	-0.119	0.685	-0.167	0.693	0.074	0.889
Serum metabolic parameters						
Δ Glucose (mg/dL)	0.188	0.519	0.411	0.312	-0.297	0.568
Δ Triglyceride (mg/dL)	0.191	0.514	0.162	0.702	0.265	0.612
Δ AST (U/I)	0.355	0.212	0.497	0.210	-0.616	0.192
Δ ALT (U/I)	0.222	0.445	0.256	0.541	0.654	0.159
Δ FGF21 (pg/mL)	**0.591**	**0.026**	**0.718**	**0.045**	0.046	0.932
Energy and nutrients intake (/day)						
Δ Energy (kcal)	-0.117	0.690	-0.343	0.405	0.232	0.658
Δ Protein (g)	-0.230	0.428	-0.468	0.242	0.185	0.726
Δ Fat (g)	-0.247	0.394	**-0.783**	**0.022**	0.344	0.504
Δ Carbohydrate (g)	-0.045	0.878	-0.130	0.758	0.217	0.679

BAT, brown adipose tissue; BMI, body mass index; AST, aspartate aminotransferase; ALT, alanine aminotransferase; FGF21, fibroblast growth factor 21. Data are the Pearson’s correlation coefficients. Boldface indicates significance.

## Discussion

The present study revealed that the single intake of beta-conglycinin did not increase serum FGF21 level, and the serum FGF21 level decreased during a 2-h rest in young men. In addition, a 2-week RCT showed that serum FGF21 concentration and BAT activity were not increased by beta-conglycinin intake. In contrast, the Δ baseline FGF21 level significantly correlated with the Δ BAT activity. These results suggested that the circulating FGF21 concentration was not affected by a single and short-term intake of beta-conglycinin, and basal FGF21 concentration was associated with human BAT activity. Since the significant correlation between Δ baseline FGF21 level and Δ BAT activity was only observed in the beta-conglycinin group, it may be suggested that soy protein intake may modulate BAT activity through FGF21.

It was reported that temporary FGF21 secretion was induced by 6-h fasting in rodents, but 1-week fasting was needed to increase serum FGF21 concentration in humans [30]. The results suggest that FGF21 responsiveness to fasting may differ between rodents and humans. Unlike a previous animal study [22], this human study found that single beta-conglycinin intake does not increase circulating FGF21 level. This might be partly caused by the different FGF21 responsiveness that has been described in fasting conditions [30]. In addition, a human study reported that circulating FGF21 concentration followed a diurnal rhythm, reaching its peak in the early morning and declining after breakfast [31]. Thus, there is a possibility that different circadian rhythms and feeding patterns may lead to different outcomes: rodents are nocturnal animals and fed ad libitum in the laboratory, while humans are diurnal animals with regular meal times. Since this study started in the morning (9:00 AM), with the subjects in starvation to examine how the beta-conglycinin intake would affect FGF21 serum levels, the observed effects may be stronger than the ones caused by beta-conglycinin intake in humans. Moreover, hepatic FGF21 secretion in mice fed beta-conglycinin was elicited by methionine imbalance because of the lower methionine composition of beta-conglycinin [22], whereas the FFQg showed that the subjects of this study had consumed various protein sources before the days of beta-conglycinin intake (data not shown). It is difficult to assume that the dietary protein source is restricted only to beta-conglycinin in humans, and thus the methionine imbalance may not occur frequently. For the above mentioned reasons, it may be difficult to evaluate the single beta-conglycinin intake effect on FGF21 levels in humans.

In contrast, the RCT showed that serum FGF21 levels in the both groups were unchanged during the 2-h cold exposure. The blood sampling in the RCT was performed under fasting condition, and thus dietary suppression of circulating FGF21 level was linked to unchanged serum FGF21 levels during the 2-h rest. In addition, because it was reported that FGF21 secretion from BAT was stimulated by cold exposure in mice [32], the decrease in serum FGF21 concentration by the circadian rhythm might be suppressed by both fasting and the cold exposure in this study. Our RCT results revealed that Δ BAT activity was correlated with Δ baseline FGF21 level only in the beta-conglycinin group. The serum FGF21 levels were decreased, unchanged, or increased in the subjects of the beta-conglycinin group. This result suggested that the response of circulating FGF21 level to beta-conglycinin intake was not consistent. However, the mechanism remains unknown. Further studies will be needed to detail the effect of beta-conglycinin intake on FGF21 metabolism in BAT.

The Δ BAT activity was negatively correlated with fat intake only in the beta-conglycinin group, and thus there is a possibility that dietary fat intake influences BAT activity. To our knowledge, it has not been reported whether dietary fat restriction modulates BAT thermogenesis. Since the FFQg questionnaire only inquired about habitual dietary intake, the effects of fat intake should be investigated in further research using a stricter experimental design. We confirmed no significant correlation between basal levels of serum FGF21 and dietary fat intake in all subjects (*r* = 0.141, *P* = 0.631) and in the beta-conglycinin group (*r* = − 0.472, *P* = 0.238). Therefore, the dietary fat intake did not affect baseline FGF21 level and the relationship to BAT activity. The sample size of this study was small, making it difficult to identify the individual differences. Large-scale studies will be needed to explore the determining factors of the relationship between BAT activity and FGF21 in the future.

This study has several limitations including small sample size. Young healthy subjects participated in the RCT, and thus the therapeutic efficacy with regards to obesity and metabolic health has not been addressed. In addition, although the RCT performed a short-term intervention because 2 weeks are enough to induce BAT adaptation [4, 6], the confounding factors of chronic elevated FGF21 levels, such as changes in body fat distribution, have also not been addressed [33, 34]. The subjects had a relatively lower BAT activity. Indeed, the average supraclavicular–chest skin temperature was 0.8 °C, while BAT-positive subjects were defined as having approximately > 1.0°C in a previous study [27]. It is necessary to examine the long-term effects of beta-conglycinin intake on BAT activity and to identify the related variables.

## Conclusions

Single intake of beta-conglycinin does not increase serum FGF21 levels, indicating that human FGF21 responsiveness is different from rodents. Moreover, although our RCT study revealed that 2-week beta-conglycinin intake did not affect serum FGF21 concentration, the changes in basal changes in FGF21 level and BAT activity were significantly and positively correlated. This result supports the importance of FGF21 in human BAT thermogenesis. Uncovered factors of human BAT activity remain, which may yield new insights into metabolic homeostasis. Therefore, it is necessary to provide novel evidence in future studies.

## Data Availability

The datasets analyzed during the current study are available from the corresponding author on reasonable request.

## Abbreviations

ALT: Alanine aminotransferase
AST: Aspartate aminotransferase
BAT: Brown adipose tissue
BMI: Body mass index
ELISA: Enzyme-linked immunosorbent assay
FFQg: Food frequency questionnaire based on food groups
FGF21: Fibroblast growth factor 21
RCT: Randomized controlled trial
